# Influence of distance to health facilities on clinical breast cancer screening behaviour among women in five sub-Saharan African countries

**DOI:** 10.1186/s12889-023-15782-w

**Published:** 2023-05-19

**Authors:** Isaac Y. Addo, Evelyn Acquah, Castro Ayebeng, Kwamena S. Dickson

**Affiliations:** 1 Centre for Social Research in Health, The University of New South Wales, Sydney, Australia; 2 Centre for Health Policy and Implementation Research, Institute of Health Research, University of Health and Allied Sciences, Ho, Ghana; 3 Department of Population and Health, University of Cape Coast, Cape Coast, Ghana

**Keywords:** Breast cancer, Malignancy, Breast screening, Breast check, Breast screen, Socio-demographic characteristics

## Abstract

**Background:**

Regular breast screening is one of the most effective ways to detect early signs of breast cancer but travel distance to cancer-diagnostic facilities can affect breast screening attendance. Yet, limited studies have examined the impact of distance to cancer-diagnostic facilities on clinical breast screening behaviour among women in sub-Saharan Africa (SSA). This study examined the influence of travel distance to a health facility on clinical breast screening behaviour in five SSA countries: Namibia, Burkina Faso, Cote D’Ivoire, Kenya, and Lesotho. The study further assessed variations in clinical breast screening behaviour across diverse socio-demographic characteristics of women.

**Methods:**

A sample of 45,945 women was drawn from the most recent Demographic and Health Surveys (DHS) for the included countries. The DHS uses 2-stage stratified cluster sampling to select nationally representative samples of women (15–49) and men (15–64) via a cross-sectional design. Proportions and binary logistic regression were used to examine associations between the women’s socio-demographic characteristics and breast screening attendance.

**Results:**

The overall proportion of survey participants who underwent clinical breast cancer screening was 16.3%. Travel distance to a health facility had a significant (p < 0.001) impact on clinical breast screening behaviour as 18.5% of participants who self-reported distance as “not a big problem” attended clinical breast screening compared to 10.8% who self-reported distance as “a big problem”. The study further found that various socio-demographic factors were significantly associated with breast cancer screening uptake, including age, education level, media exposure, wealth status, parity, contraceptive use, health insurance coverage, and marital status. The multivariate analysis controlling for other factors confirmed the strong association between distance to health facilities and screening uptake.

**Conclusions:**

The study found that travel distance is a significant factor affecting clinical breast screening attendance among women in the selected SSA countries. Furthermore, the likelihood of breast screening attendance varied depending on different women’s characteristics. It is crucial to prioritise breast screening interventions, particularly among the disadvantaged women identified in this study, to achieve maximum public health benefits.

## Background

Breast cancer is a significant global health issue and the leading malignancy in Africa [1]. In 2018 alone, an estimated 168, 690 breast cancer cases and 74, 072 breast cancer-related deaths were reported in Africa [2]. In 2020, breast cancer was the top malignancy among females in Africa, with an estimated 186,598 cases and 85,787 deaths [3]. If left unaddressed, breast cancer incidence in sub-Saharan Africa (SSA) is projected to double by 2040 due to factors, such as rapid population ageing, population growth, and changes in fertility patterns [4]. These changes include women having fewer children and delaying their age at first childbearing, which are known risk factors for breast cancer, according to the World Health Organisation [4].

Evidence further shows that breast cancer causes over one-third of annual deaths among women in SSA [4, 5]. Breast cancer can appear in various forms and normally as a painless lump in the breast indicating the need for timely and regular screening at a recognised cancer-diagnostic facility and with a qualified health practitioner [1]. Women with a lump or thickening in the breast are advised to visit a recognised health facility within the first two months of detection [1]. Given the importance of early screening, prevention, and treatment, the WHO established the Global Breast Cancer Initiative (GBCI) in 2021, which aimed to reduce breast cancer incidence by 2.5% annually through stakeholder collaborations and key strategies, including health promotion, early detection, and timely diagnosis and treatment [5].

There are several commonly used methods for breast cancer screening, including mammography, clinical breast examination (CBE), breast self-examination (BSE), magnetic resonance imaging (MRI), and breast ultrasound [6–8]. Mammography is the most widely used screening method, especially for women over 50 years [9]. CBE is a physical examination of the breast by a healthcare professional and can be used as a standalone screening method or in combination with mammography [7]. BSE is a simple screening method that women can perform on themselves to detect any changes in their breast tissue [10]. MRI is not recommended as a routine screening method for the general population, but it may be used in combination with mammography for high-risk women or those with dense breast tissue [11]. Breast ultrasound may be used in combination with mammography or MRI for women with dense breast tissue or to evaluate suspicious findings on mammography or CBE [6, 11].

The appropriate age for breast cancer screening is a topic of ongoing debate, with various recommendations from different organisations. The American Cancer Society recommends annual screening from age 45–54, with the option to start at age 40. [12]. Cancer Australia provides free screening to women aged 40–49 and over 75 years, while women aged 50–74 years are recommended to attend the BreastScreen Australia Program for free two-yearly mammograms [13]. The WHO suggests organised screening programs for women aged 40–49 years in well-resourced settings [14]. Most guidelines recommend annual or biennial mammographic screening for women aged 40–74 years for average-risk populations, and more frequent screening for high-risk groups [15]. Some studies suggest CBE may be more effective than mammography in detecting breast cancer in younger women and those with dense breast tissue [6, 16, 17]. The absence of complete agreement among organisations and settings regarding breast cancer screening recommendations highlights the importance of further research to improve age-specific screening guidelines while considering individual risk factors, including race, ethnicity, and variations in breast tissue mass, among other factors.

In this present study, we examined the influence of travel distance to a health facility on clinical breast screening behaviour in SSA using recently released Demographic and Health Survey (DHS) data for five countries: Namibia, Burkina Faso, Cote D’Ivoire, Kenya, and Lesotho. We hypothesised that women who self-identified travel distance to a health facility as a big problem, regardless of their location, will be less likely to visit a health facility for breast screening compared to those who regarded travel distance to a health facility as ‘not a big problem’. We, therefore, examined women’s breast screening behaviour by those who self-identified distance to a health facility as a ‘big problem’ and those who self-identified distance to a health facility as ‘not a big problem’ and across various socio-demographic characteristics. This method represents an improvement over previous approaches and can inform various stakeholders, such as the WHO’s Global Breast Cancer Initiative (GBCI), clinicians, policymakers, cancer foundations, cancer registries, governments, and researchers in SSA. It is, however, worth noting that our study was subject to a constraint resulting from the age range of female participants, which was limited to individuals aged 15–49, as dictated by the available DHS data. This type of constraint is a frequent challenge in research, where data availability imposes limits on the study’s scope. Consequently, the study outcomes may not be fully representative of the larger population, including other age cohorts or genders beyond the female demographic.

## Methods

### Data source

The present study analysed data from the most recent Demographic and Health Surveys (DHS), which collects comprehensive information on various topics such as fertility, breast cancer, cervical cancer, infant and child mortality, and maternal and child care. The DHS employs a two-stage stratified cluster sampling approach to select nationally representative samples of women in their reproductive age groups (15–49 years) and men aged 15–64, using a cross-sectional study design as the conventional method. The study sample was drawn from only five sub-Saharan African (SSA) countries, namely Namibia, Burkina Faso, Cote D’Ivoire, Kenya, and Lesotho, and comprised a total of 45,945 women who had data on the outcome of interest. We obtained approval from the MEASURE DHS after presenting our concept note, and the dataset is accessible via https://dhsprogram.com/methodology/survey/surveydisplay-491.cfm. To ensure transparent reporting, we followed the Strengthening the Reporting of Observational Studies in Epidemiology (STROBE) statement while conducting the study and writing the manuscript [18]. The DHS’s comprehensiveness and representativeness make it an ideal data source for our study’s variables of interest.

### Study variables and measurements

#### Outcome variable

In this study, the outcome variable of interest was “breast cancer screening,“ which was assessed using the question, “Have you ever screened for breast cancer?“ Respondents’ answers were recorded as either “no = 0” or “yes = 1.“ Consistent with previous research utilising DHS data, we employed the binary response of breast cancer screening (yes/no) as the dependent variable in our analysis. The survey offered three response options for screening modalities: clinical breast examination, ultrasound, and mammography. More detailed information on breast cancer screening questionnaires can be found in previous studies [19, 20].

#### Explanatory variables

Thirteen explanatory variables were used in agreement with both theoretical and empirical literature [2, 21, 22]. The primary explanatory variable in this study was the distance to the nearest health facility, categorised as either a big problem (0) or not a big problem (1). In addition to this variable, the study included twelve other explanatory variables, which were chosen based on both theoretical and empirical literature [2, 21, 22]. These variables were: women’s age, place of residence (urban or rural), level of education, frequency of reading newspapers or magazines, frequency of listening to the radio, frequency of watching television, wealth status, parity (number of children), contraceptive use, coverage by health insurance, marital status, and country of residence (Burkina Faso = 1, Cote d’Ivoire = 2, Kenya = 3, Lesotho = 4, Namibia = 5) (see Table 1). Women’s age was categorised into seven groups (15–19 = 1, 20–24 = 2, 25–29 = 3, 30–34 = 4, 35–39 = 5, 40–44 = 6, 45–49 = 7), while level of education was categorised into four groups (no education = 0, primary = 1, secondary = 2, higher = 3). The frequency of reading newspapers or magazines, listening to radio, and watching television were each categorised into three groups (not at all = 0, less than once a week = 1, at least once a week = 2). Wealth status was assessed using a five-point scale (poorest = 1, poorer = 2, middle = 3, richer = 4, richest = 5), while parity was categorised into four groups based on the number of children (1–2 = 1, 3–5 = 2, 6–8 = 3, 9 + = 4). Contraceptive use was categorised as either not using (0) or using (1), while coverage by health insurance was categorised as either no (0) or yes (1). Finally, marital status was categorised into six groups (never in union = 1, married = 2, living with partner = 3, widowed = 4, divorced = 5, separated = 6). These explanatory variables were selected based on their potential influence on the outcome of interest and their relevance to the research question. The details of the variable categorisation are presented in Table 1.

### Data analysis

The present study employed both descriptive and inferential analyses to investigate the association between breast cancer screening and various explanatory variables. The descriptive analysis involved bivariate analysis of the country variables and the outcome variables. Additionally, it provided the frequency and proportion of background characteristics by outcome variables. To further examine the significant association between the outcome variable (breast cancer screening) and the respondents’ explanatory variables, a binary logistic regression model was utilised in a multivariate analysis. Due to the dichotomous nature of the outcome variable, two binary logistic regression models were employed. The first model investigated the association between the main independent factor, i.e., distance to the health facility, and the outcome variable. The second model analysed the direct relationship between distance to the health facility and the outcome variable, while adjusting for other relevant independent variables that were known to influence the outcome variable. The hierarchical nature of the Demographic and Health Survey (DHS), which involves respondents being layered within survey clusters, has the potential to bias standard errors. Therefore, the Huber-White technique was utilised to derive robust standard errors, as suggested by Ayebeng, et al. [23]. A multicollinearity test was performed on each variable, and the results indicated that the variables in the models had a mean-variance inflation factor (VIF) of 2.34. A VIF score greater than 10 suggests the presence of multicollinearity [24]. Using a 95% confidence interval, the adjusted odds ratios for each variable were determined. The data were handled and analysed using Stata (Version 17). The outcomes were sample-weighted to address any under- or over-sampling of participants from the total population.

## Results

### Survey years and proportions of women who screened for breast cancer by country

Table 1 shows that the overall proportion of respondents who underwent breast cancer screening was 16.3%. However, the proportion of women screened for breast cancer varied across the five different SSA countries included in the study. Specifically, the lowest rate of breast cancer screening was found in Cote D’Ivoire, with only 5.2% of respondents undergoing screening. In contrast, the highest rate of screening was observed in Kenya, with 25.5% of respondents being screened.

**Table 1:** Country, survey year, and proportion screened for breast cancer

Country	Survey year	Frequency	Proportion of breast cancer screened
Burkina Faso	2010	9,451	7.5
Cote D’Ivoire	2011–2012	6,320	5.2
Kenya	2014	14,552	25.5
Lesotho	2014	6,558	9.7
Namibia	2013	9,064	23.1
**Total**		**45,945**	**16.3**

### Socio-demographic characteristics of women who screened for breast cancer

Table 2 displays the socio-demographic characteristics of the women who underwent breast cancer screening. Out of the respondents who identified distance to health facilities as a significant obstacle, 10.8% underwent breast cancer screening. This percentage is lower than that of those who did not identify distance as a significant problem, of whom 18.5% underwent screening. The majority of respondents who underwent screening were aged 40–44 years (21.1%), lived in urban areas (21.1%), had higher levels of education (40.2%), belonged to the richest wealth index category (23.7%), were divorced (26.9%), had given birth to 3–5 children (18.4%), were covered by health insurance (42.3%), and used contraceptives (22.0%). In contrast, those who did not undergo screening included respondents aged 15–19 years (7.9%), living in rural areas (12.1%), having no education (5.4%), belonging to the poorest wealth index category (7.6%), never in union (13.7%), gave birth to 9 or more children (7.5%), were not covered by health insurance (13.2%), and were not using contraceptives (12.7%).

Furthermore, a higher percentage of women who read newspapers or magazines at least once a week (28.4%), listened to the radio at least once a week (20.0%), and watched television at least once a week (22.5%) underwent breast cancer screening. Conversely, those who never read newspapers nor magazines (11.6%), never listened to the radio (9.5%), and never watched television (11.7%) had lower rates of screening.

The Chi-square test revealed statistically significant differences among all the independent variables used in the analysis, as indicated by the corresponding p-values.

**Table 2:** Socio-demographic characteristics and proportion of women screened for breast cancer

Variable	Frequency (N = 45,945)	Proportion screened for breast cancer	X^2^ (p-value)
***Distance to health facility***			381.9 (p < 0.001)
Big problem	13,524	10.8	
Not a big problem	32,421	18.5	
***Age***			671.3 (p < 0.001)
15–19	8,579	7.9	
20–24	8,915	13.8	
25–29	8,467	18.8	
30–34	6,818	19.2	
35–39	5,519	19.5	
40–44	4,269	21.1	
45–49	3,378	20.2	
***Place of residence***			442.8 (p < 0.001)
Urban	21,128	21.1	
Rural	24,817	12.1	
***Level of education***			2.0e + 03 (p < 0.001)
No education	10,081	5.4	
Primary	14,980	14.2	
Secondary	17,397	19.5	
Higher	3,487	40.2	
***Frequency of reading newspapers or magazine***			1.2e + 03 (p < 0.001)
Not at all	28,441	11.6	
Less than once a week	8,685	19.1	
At least once a week	8,819	28.4	
***Frequency of listening to radio***			611.6 (p < 0.001)
Not at all	10,547	9.5	
Less than once a week	9,100	13.1	
At least once a week	26,298	20.0	
***Frequency of watching television***			643.6 (p < 0.001)
Not at all	21,513	11.7	
Less than once a week	6,587	14.0	
At least once a week	17,845	22.5	
***Wealth status***			732.0 (p < 0.001)
Poorest	6,619	7.6	
Poorer	7,614	12.2	
Middle	8,268	14.0	
Richer	10,128	17.0	
Richest	13,316	23.7	
***Parity***			186.9 (p < 0.001)
1–2	27,827	16.5	
3–5	15,586	18.4	
6–8	4,505	10.8	
9+	1,027	7.5	
***Contraceptive use***			673.2 (p < 0.001)
Not using	28,318	12.7	
Using	17,627	22.0	
***Covered by health insurance***			2.3e + 03 (p < 0.001)
No	41,181	13.2	
Yes	4,764	42.3	
***Marital status***			175.7 (p < 0.001)
Never in union	15,534	13.7	
Married	22,265	17.5	
Living with partner	4,172	15.7	
Widowed	1,507	15.9	
Divorced	603	26.9	
Separated	1,864	21.4	

Notes: “N” represents the sample size or the total number of observations, X² represents “Chi-squared”, and the p-value represents the significant levels

### Factors associated with breast cancer screening

Table 3 displays the results of the multivariate analysis examining the factors associated with breast cancer screening uptake among the included women. The analysis revealed that various demographic, socioeconomic, and health-related factors were significantly associated with breast cancer screening. Specifically, distance to health facility, age, place of residence, level of education, frequency of reading newspapers and magazines, frequency of listening to the radio, wealth status, parity, contraceptive use, health insurance coverage, and marital status were all found to have a significant association with breast cancer screening uptake.

**Table 3:** Multivariate analysis of factors associated with breast cancer screening

Variable	Model 1 OR (95% CI)	Model 2 AOR (95% CI)
***Distance to health facility***		
Big problem	Ref	Ref
Not a big problem	1.84***(1.73, 1.95)	1.30***(1.21, 1.39)
***Age***		
15–19		Ref
20–24		1.69 *** (1.43, 179)
25–29		2.05 ***(1.83, 2.30)
30–34		2.24 ***(1.98, 2.53)
35–39		2.56 ***(2.25, 2.91)
40–44		3.01 ***(2.63, 3.45)
45–49		3.05***(2.63, 3.52)
***Place of residence***		
Urban		Ref
Rural		0.84***(0.78, 0.90)
***Level of education***		
No education		Ref
Primary		2.23 ***(2.02, 2.47)
Secondary		2.67 ***(2.40, 2.98))
Higher		3.96 ***(3.44, 4.55)
***Frequency of reading newspapers or magazine***		
Not at all		Ref
Less than once a week		1.15 ***(1.07, 1.24)
At least once a week		1.41 ***(1.30, 1.52)
***Frequency of listening to radio***		
Not at all		Ref
Less than once a week		1.03 (0.93, 1.13)
At least once a week		1.31 ***(1.21, 1.42)
***Frequency of watching television***		
Not at all		Ref
Less than once a week		0.92(0.84, 1.01)
At least once a week		1.05(0.98, 1.15)
***Wealth status***		
Poorest		Ref
Poorer		1.19 **(1.07, 1.33)
Middle		1.20 **(1.08, 1.34)
Richer		1.15 **(1.02, 1.29)
Richest		1.07(0.94, 1.21)
***Parity***		
1–2		Ref
3–5		1.04(0.96, 1.12)
6–8		0.80 ***(0.71, 0.91)
9+		0.66 **(0.52, 0.84)
***Contraceptive use***		
Not using		Ref
Using		2.36***(2.18, 2.55)
***Covered by health insurance***		
No		Ref
Yes		2.36 ***(2.18, 2.55)
***Marital status***		
Never in union		Ref
Married		1.26 ***(1.16, 1.36)
Living with partner		1.18 **(1.06, 1.32)
Widowed		1.00(0.85, 1.78)
Divorced		1.59 ***(1.29, 1.96)
Separated		1.50 ***(1.31, 1.72)

**Notes**: *P < 0.05 **p < 0.01 ***p < 0.001, Ref; Reference category, OR = odds ratio, AOR = adjusted odds ratio, 95% CI: 95% confidence interval

The results of model 1 demonstrated that women who perceived distance to a health facility as not being a big problem had a significantly higher likelihood of screening for breast cancer than those who perceived it as a big problem. Specifically, the odds of screening for breast cancer were found to be 1.84 times higher among women who perceived distance to a health facility as not being a big problem compared to those who identified it as a big problem. In order to determine the effect of distance to a health facility on breast cancer screening uptake while accounting for other important factors, a multivariate analysis was conducted. Model 1 showed that women who did not view distance to a health facility as a big problem were 1.84 times more likely to undergo breast cancer screening than those who did view it as a big problem.

To further investigate this association, Model 2 was developed, which controlled for other relevant factors that may influence breast cancer screening, including age, level of education, place of residence, exposure to media, wealth status, parity, contraceptive use, subscription to health insurance, and marital status. After controlling for these factors, the statistically significant association between distance to a health facility and breast cancer screening uptake remained strong, with women who did not view distance to a health facility as a big problem being 1.30 times more likely to undergo breast cancer screening compared to those who did view it as a big problem.

Table 3 shows the results of the multivariate analysis examining the association between various factors and breast cancer screening uptake. The control variables included age, level of education, place of residence, exposure to media (reading newspaper/magazine, listening to the radio, and watching television), wealth status, parity, contraceptive use, health insurance coverage, and marital status. The analysis revealed a statistically significant association between these factors and breast cancer screening uptake.

Age was positively associated with breast cancer screening uptake, with women aged 45–49 years having a higher likelihood of screening (AOR = 3.05, CI = 2.63,3.52). Educational attainment was also positively associated with screening uptake, with women who had completed formal education having a higher likelihood of screening (AOR = 3.96, CI = 3.44, 4.55) than those with no formal education.

Marital status was also associated with screening uptake, with separated women having a higher likelihood of screening (AOR = 1.50, CI = 1.31,1.72) than those who had never married. Exposure to media, specifically reading newspapers or magazine (AOR = 1.41, CI = 1.30,1.52) and listening to the radio at least once a week (AOR = 1.31, CI = 1.21,1.42), was positively associated with screening uptake.

Wealth status was positively associated with screening uptake, with women in the middle (AOR = 1.20, CI = 1.08,1.34) and richer (AOR = 1.15, CI = 1.02,1.29) wealth index having a higher likelihood of screening than those in the poorest wealth index. Parity was negatively associated with screening uptake, with women who had given birth to 6–8 (AOR = 0.80, CI = 0.71,0.91) and nine or more (AOR = 0.66, CI = 0.52,0.84) children being less likely to screen than those who had 1–2 children.

Contraceptive use (AOR = 2.36, CI = 2.18,2.55) and health insurance coverage (AOR = 2.36, CI = 2.18,2.55) were positively associated with screening uptake. Place of residence was also associated with screening uptake, with women in rural areas being less likely to screen (AOR = 0.84, CI = 0.78,0.90) than those in urban areas.

Overall, the multivariate analysis showed that distance to health facility, age, level of education, place of residence, exposure to media, wealth status, parity, contraceptive use, health insurance coverage, and marital status were all significantly associated with breast cancer screening uptake.

## Discussion

This paper examined the influence of distance to a health facility on breast cancer screening behaviour in sub-Saharan Africa (SSA) using recently released Demographic and Health Survey data for five countries: Burkina Faso, Cote D’Ivoire, Kenya, Lesotho, and Namibia. The study was motivated by the need to understand the drivers of breast cancer screening behaviour, with a particular interest in the extent to which travel distance to healthcare facilities influences women’s likelihood of presenting for breast screening in SSA.

Our findings revealed a breast cancer screening rate of approximately 16%, which is higher than a previous rate of 12.9% reported in similar settings by Ba, et al. [20]. Furthermore, we found a relatively higher proportion of breast cancer screening across the sampled countries- (Burkina Faso (7.5%), Cote D’Ivoire (5.2%), Kenya (%) and Namibia (25.3%) compared to the rates found by Ba, et al. [20]. However, it is important to acknowledge that our study focused on assessing factors associated with screening in a younger population, which may limit the generalisability of our findings to an older screening-eligible population. The low breast cancer screening rate observed in our study may have been influenced by the fact that some participants may not be of the recommended screening age. Therefore, we recommend caution when applying our study findings to older populations. Further research is needed to assess the factors associated with breast screening behaviour in older women. By conducting further research, we can better understand the factors influencing screening behaviour in all age groups and ensure that screening programs are effective for all women.

Out of the women that self-identified distance to a health facility as a big problem, only 10.8% screened for breast cancer compared to 18.5% of those who indicated that distance to a health facility was not a big problem. This finding indicates that travel distance problems discourage the likelihood of breast screening attendance among women in the five countries, and the opposite might be true when the distance to health facilities is not a significant problem. Even after controlling for other relevant socio-demographic factors known to have influenced breast cancer screening, the role of distance to health facilities was statistically significant. This observation calls for a need to consider disadvantaged women affected by travel distance when devising breast screening interventions to promote a more positive breast cancer screening behaviour. Nevertheless, it is important to acknowledge that we used self-identification of distance to health facilities as a problem as our exposure variable, rather than actual distance. Given that this measure is not very specific, we recognise the importance of interpreting our findings cautiously.

We also found that age, place of residence, level of education, frequency of reading newspapers and magazines, frequency of listening to the radio, wealth status, parity, contraceptive use, health insurance, and marital status had a significant association with the likelihood of breast cancer screening attendance. This observation shows similarity to a previous study in Nigeria - Angela, Adewole and Iyanuoluwa [21]. As can be seen in this paper, age was significantly associated with breast cancer screening, with women aged 45–49 years having a higher likelihood of screening for breast cancer compared to those aged 15–19 years in both models. The age disparities in breast cancer screening could be related to the existing breast cancer screening guidelines in these countries which may favour older women more than younger ones. For instance, a systematic review of 23 guidelines issued between 2010 and 2021 in 11 countries or regions highlighted similar recommendations for systematic mammography screening for women aged 40 years and over [25] and this may have influenced medical practices and recommendations from physicians leading to higher odds of breast screening among older women than younger ones. This finding also corroborates other findings in South Africa [26–28].

The findings underscore the peculiar importance of formal education in breast cancer screening behaviour. We observed that women with higher levels of formal education were more likely to utilise breast cancer screening opportunities compared to those who had no formal education. This result substantiates similar studies conducted previously [29, 30]. For example, Agyemang, et al. [31] made a parallel observation that older Ghanaian women with higher educational levels were more likely to attend breast cancer screening compared to their counterparts with no formal education. A plausible explanation is that more educated women may have been exposed to lessons and literature that might have enhanced their understanding of the implications of breast cancer screening and hence are more likely to utilise such services.

The study revealed disparities in breast screening behaviour across women’s places of residence. Thus, women living in rural areas were less likely to utilise breast cancer screening than those in urban settings. The lower likelihood of breast cancer screening attendance in rural areas is not surprising and could be due to disparities in access to healthcare or a lack of adequate hospitals and other healthcare facilities to offer breast cancer screening services. Previous research has found similar results in Namibia [29], and South Africa [32].

Another finding worth commenting on is the higher odds of breast cancer screening among women who were exposed to the media (e.g., frequency of reading magazines and newspapers, and frequency of listening to the radio) at least once a week compared to those who were not exposed at all. Intensive publicity and educative programs on various mass media platforms on the health benefits of periodic breast examinations for early detection and treatment had the potential to improve breast cancer screening behaviour among women by building on their awareness levels. This finding is consistent with previous research that revealed that most Kenyan women desired to be informed about screening activities via messages shared on local radio stations [33]. Health education on breast cancer via magazines, newspapers, and radio can enhance the uptake of breast cancer screening opportunities across the selected countries and beyond.

One striking finding is that women who subscribed to health insurance coverage were more likely to be screened for breast cancer than their counterparts without health insurance coverage. Having health insurance coverage may subsidize or fully cover the cost of breast cancer screening services which may then provide an opportunity for women to utilise the service. This finding corresponds with previous studies that also indicated that women with health insurance coverage were more likely to be screened for breast cancer [20, 34]. We found that women with middle and richer wealth indexes were more likely to be screened for breast cancer compared to the poorest women. A possible reason could be that women with strong financial backing are more likely to afford preventive care services including breast cancer screening which may not be the case for women of the lowest economic status.

Regarding women’s birth parity, this study revealed that those with higher parity had lower odds of screening for breast cancer than their counterparts with lower parity. This may imply that older women with high birth parity might be less concerned with considerations such as finding a partner which has shown to be a significant factor influencing breast screening behaviours among young women in some parts of sub-Saharan Africa [14].

Another important observation is the positive association between contraceptive use and the likelihood of screening for breast cancer. A possible reason is that women who used contraceptive services might have been more familiar with reproductive health services of which breast cancer screening might have been included. Moreover, the study revealed disparities in screening for breast cancer among different marital statuses. Compared to never-married women, women who were married (married and living together) and formerly married (separated and divorced) were more likely to screen for breast cancer. This present finding corroborates findings in previous evidence [20]. Perhaps, sexual unions might have been a supportive tool for preventative healthcare services such as early breast cancer screening.

## Strengths and limitations of the study

This study utilised a nationally representative dataset from five sub-Saharan African countries, providing generalisable findings for the sampled countries. The validity and reliability of our findings are strengthened by the use of the DHS dataset, which has been validated in multiple instances. However, there are several limitations worth mentioning. Firstly, the study is cross-sectional, which limits the ability to establish causality between the explanatory and outcome variables. Additionally, the use of secondary data prevents the ascertainment of possible cultural factors that may influence the association between breast cancer screening attendance and distance to health facilities, as well as socio-demographic factors. Furthermore, social desirability and recall bias may have affected the data collection process, and the concept of distance to health facilities may not be relevant in situations where health facilities lack the capacity and resources to screen for cancer. We acknowledge that our assessment of distance problems relied on self-reported identification, which may have been influenced by other factors beyond physical distance, such as transportation access, socioeconomic status, and personal perceptions. These limitations may have impacted the accuracy and specificity of our findings, highlighting the need for further research to directly measure distance and explore other factors contributing to distance-related barriers to breast cancer screening.

Moreover, our study population only included women aged 15 to 49 years, which is younger than the recommended mammography screening age in most settings. However, we considered this age range due to the recognition that breast cancer can affect women of all ages, and early detection is crucial for optimal outcomes. Also, our study was limited to female participants aged 15–49 due to constraints imposed by the available DHS data. It is also worth noting that our findings may be affected by self-breast checks or Breast Self-Examination (BSE). Although our study population may not be representative of the 50 + age group, our findings can still offer insights into improving breast cancer screening rates for women of all ages, highlighting the need for interventions to promote breast cancer screening while considering the factors that place many women at a disadvantage, including long distances to health facilities. Additionally, we recommend that the included SSA countries should channel more commitment and efforts through advocacy and education via the media (newspaper/magazine, and radio) to improve the uptake of breast cancer screening programs and their associated public health benefits.

## Conclusions

In this study, we examined the association between distance to health facilities and breast cancer screening behaviour in five sub-Saharan African countries, namely Namibia, Burkina Faso, Cote D’Ivoire, Kenya, and Lesotho. Our findings demonstrate that travel distance to health facilities is associated with a lower rate of breast cancer screening. Moreover, we identified several factors that are significantly associated with breast cancer screening behaviour, including age, education level, media exposure, wealth status, parity, contraceptive use, health insurance coverage, and marital status. To promote breast cancer screening among women in these countries, it is crucial to address the barriers to healthcare access, particularly the long distances to health facilities. We recommend that these countries prioritise advocacy and education through the media (newspaper/magazine and radio) to improve awareness of breast cancer screening and its associated public health benefits. By considering the factors placing many women at a disadvantage and promoting education and awareness, we can improve the uptake of breast cancer screening programs and ultimately reduce breast cancer-related morbidity and mortality.

## Data Availability

The dataset(s) supporting the conclusions of this article is(are) available in the DHS repository at: http://dhsprogram.com/data/available-datasets.cfm.

## Abbreviations

AOR: adjusted Odds Ratio
CI: Confidence Interval
DHS: Demographic and Health Surveys
SSA: Sub-Saharan African
VIF: variance inflation factor
WHO: World Health Organisation
